# tDCS-Induced Memory Reconsolidation Effects and Its Associations With Structural and Functional MRI Substrates in Subjective Cognitive Decline

**DOI:** 10.3389/fnagi.2021.695232

**Published:** 2021-07-26

**Authors:** Lídia Vaqué-Alcázar, Lídia Mulet-Pons, Kilian Abellaneda-Pérez, Cristina Solé-Padullés, María Cabello-Toscano, Dídac Macià, Roser Sala-Llonch, Nuria Bargalló, Javier Solana, Gabriele Cattaneo, José M. Tormos, Alvaro Pascual-Leone, David Bartrés-Faz

**Affiliations:** ^1^Department of Medicine, Faculty of Medicine and Health Sciences, Institute of Neurosciences, University of Barcelona, Barcelona, Spain; ^2^Institut de Recerca Biomèdica August Pi i Sunyer (IDIBAPS), Barcelona, Spain; ^3^Guttmann Institute, Badalona, Spain; ^4^Department of Biomedicine, Faculty of Medicine and Health Sciences, Institute of Neurosciences, University of Barcelona, Barcelona, Spain; ^5^Consorcio Centro de Investigación Biomédica en Red (CIBER) de Bioingeniería, Biomateriales y Nanomedicina (CIBER-BBN), Barcelona, Spain; ^6^Centre de Diagnòstic per la Imatge Clínic, Hospital Clínic de Barcelona, Barcelona, Spain; ^7^Harvard Medical School, Hinda and Arthur Marcus Institute for Aging Research and Deanna and Sidney Wolk Center for Memory Health, Hebrew SeniorLife, Boston, MA, United States

**Keywords:** reconsolidation, episodic memory, transcranial direct current stimulation, magnetic resonance imaging, subjective cognitive decline

## Abstract

Previous evidence suggests that transcranial direct current stimulation (tDCS) to the left dorsolateral prefrontal cortex (l-DLPFC) can enhance episodic memory in subjects with subjective cognitive decline (SCD), known to be at risk of dementia. Our main goal was to replicate such findings in an independent sample and elucidate if baseline magnetic resonance imaging (MRI) characteristics predicted putative memory improvement. Thirty-eight participants with SCD (aged: 60–65 years) were randomly assigned to receive active (*N* = 19) or sham (*N* = 19) tDCS in a double-blind design. They underwent a verbal learning task with 15 words (DAY-1), and 24 h later (DAY-2) stimulation was applied for 15 min at 1.5 mA targeting the l-DLPFC after offering a contextual reminder. Delayed recall and recognition were measured 1 day after the stimulation session (DAY-3), and at 1-month follow-up (DAY-30). Before the experimental session, structural and functional MRI were acquired. We identified a group^∗^time interaction in recognition memory, being the active tDCS group able to maintain stable memory performance between DAY-3 and DAY-30. MRI results revealed that individuals with superior tDCS-induced effects on memory reconsolidation exhibited higher left temporal lobe thickness and greater intrinsic FC within the default-mode network. Present findings confirm that tDCS, through the modulation of memory reconsolidation, is capable of enhancing performance in people with self-perceived cognitive complaints. Results suggest that SCD subjects with more preserved structural and functional integrity might benefit from these interventions, promoting maintenance of cognitive function in a population at risk to develop dementia.

## Introduction

Recent conceptualizations of major brain diseases affecting older adults incorporate the idea of a long preclinical phase, where pathological brain changes have already started but individuals remain asymptomatic (Dubois et al., 2016). These preclinical individuals might experience cognitive decline that may not be readily detectable with the neuropsychological tools commonly employed in clinical settings. Within this context, the condition termed subjective cognitive decline (SCD) has been defined to refer to individuals over 60 years of age, without evidence of objective cognitive impairment on formal testing, but who report self-perception of worsening cognitive capacity (Jessen et al., 2014). SCD is commonly present in preclinical stages of neurodegenerative diseases (Parfenov et al., 2020), and is considered a risk factor for developing amnestic mild cognitive impairment (MCI) and subsequently dementia due to Alzheimer’s Disease (AD; Abdulrab and Heun, 2008; Reisberg and Gauthier, 2008).

Cognitive dysfunction is highly prevalent with advancing age (Plassman et al., 2013) and represents a major societal health problem that negatively impacts the quality of life and increases risk for dementia (Wittchen et al., 2011). Therefore, there is an urgent need to identify effective interventions to delay memory decline and promote maintenance of cognitive function. Current pharmacological approaches offer little effectiveness (Karakaya et al., 2013); thus, alternative strategies are required, including transcranial direct current stimulation (tDCS, Hsu et al., 2015; Tatti et al., 2016; Abellaneda-Pérez et al., 2019). Recent investigations reveal that tDCS applied over the left dorsolateral prefrontal cortex (l-DLPFC) could enhance verbal episodic memory performance through the modulation of memory reconsolidation process in older adults (Sandrini et al., 2014, 2019), individuals with SCD (Manenti et al., 2017) and even among MCI patients (Manenti et al., 2018). It is worth noting that memory formation processes operate on a highly dynamic fashion. In this sense, for a limited period of time after encoding, new memories remain unstable, and they are stabilized through the consolidation process. Nevertheless, consolidated memories may return to an unstable state where they might be reactivated (for instance by a reminder). This would open a time-limited window where such memories could be modified, in the so-called reconsolidation process (Forcato et al., 2014). During reconsolidation, active memory traces are vulnerable to be modified by external interventions such as tDCS, which could potentially be able to stabilize (i.e., strengthening) new memories (reviewed in Sandrini et al., 2015, 2020). This process might be possible due to tDCS’s potential to facilitate neuroplasticity mechanisms (Nitsche et al., 2003), which might be crucial during this time-limited reconsolidation window. Thus, long-term benefits from these interventions might probably be associated with plasticity-related persistent modifications in synaptic connections following tDCS (Stagg and Nitsche, 2011).

Recently, Sandrini et al. (2020) reviewed the effects of tDCS on episodic memory in aging and concluded that one of the main limitations in this field is the lack of reliability. Thus, despite the growing body of work suggesting that brain stimulation techniques might be used to induce episodic memory improvements in aging (Huo et al., 2019), many of the reported cognitive effects still need to be independently replicated and mechanistically elucidated (Sandrini et al., 2020). One of the major factors underlying this lack of reliability is the observed individual variability in response to the various non-invasive brain stimulation (NIBS) protocols (i.e., López-Alonso et al., 2014; Wiethoff et al., 2014). Potential factors contributing to such variability include magnetic resonance imaging (MRI) features; from both structural (e.g., Kim et al., 2014) and functional perspectives [e.g., connectivity profile of central brain networks such as the default-mode network (DMN); Vidal-Piñeiro et al., 2015; Abellaneda-Pérez et al., 2018; Antonenko et al., 2019].

The first goal of the present study was to corroborate whether tDCS applied over the left l-DLPFC after a contextual reminder could improve episodic memory reconsolidation in an independent cohort of SCD participants. The second objective was to investigate if MRI-based measures of structural and functional brain integrity obtained prior to the memory reconsolidation experiment would entail predictive value regarding the cognitive effects. We hypothesized that, like reported previously (Manenti et al., 2017), SCD subjects would improve verbal episodic memory performance through the application of active tDCS over l-DLPFC during reconsolidation after a reminder. In addition, we predicted that the inter-individual variability in response to tDCS could be explained by the degree of brain integrity, specifically among structures associated with either reconsolidation processes or DMN connectivity, as a key network susceptible to both the effects of aging (Andrews-Hanna et al., 2007; Staffaroni et al., 2018) and AD (Liu et al., 2014; Chen et al., 2016).

## Materials and Methods

### Participants

Seventy-eight subjects from the Barcelona Brain Health Initiative cohort (BBHI; Cattaneo et al., 2018) aged 60 or above were contacted via telephone. First, a pre-recruitment screening was conducted. Subjects were pre-selected if they answered *“agree”* or *“absolutely agree”* in at least one of the following items included in a previously online administered Patient-Reported Outcomes Measurement Information System-Cognitive Function (PROMIS-CF; Ader, 2007) questionaire: “*I had problems reasoning or recalling things”; “I had to read something several times to understand it”; “I had trouble remembering new information, like phone numbers or simple instructions”; “I had to work really hard to pay attention or I would make a mistake”; “I had trouble remembering whether I did things I was supposed to do.”* Then, pre-selected participants underwent a screening call, whereby they were asked if they felt their memory was becoming worse. If the answer was positive, subjects were invited to enroll in the study. Upon initial consent, exclusion criteria were reviewed, which contained any of the following: neurologic or psychiatric diagnosis, contraindication to NIBS (Rossi et al., 2009; Antal et al., 2017), as well as any contraindication for MRI (including claustrophobia or metal and electronic implants). After applying these criteria, a total of 51 participants were selected for the first visit to our center, where a battery of neuropsychological tests assessing the main cognitive domains was administered (for further details see Supplementary Material Section 1.1). To ensure that all participants had a normal cognitive profile, final inclusion criteria were Mini-Mental State Examination (MMSE) score above 25 (Mitchell, 2009) and performances on neuropsychological tests no more than 1.5 standard deviations (SD) below normative scores adjusted by age and years of education (Peña-Casanova et al., 2009). In addition, clinical data were obtained, and we included participants if they had a previous diagnosis of hypertensive, diabetic, or hyperlipidemic conditions, and they were being treated with antihypertensive, antidiabetic, cholesterol-lowering agents, respectively. Six volunteers were excluded due to abnormal performance in neuropsychological assessment, two for brain MRI abnormalities (aneurysm and meningioma) and four due to methodological problems during the experimental design. Finally, one participant decided to withdraw from the study. Overall, 38 tDCS naïve individuals with mean age of 62.29 years (*SD* = 1.56) were finally included.

All study procedures were approved by the Institutional Review Board (IRB 00003099—amendment at the University of Barcelona) and *Comité d’Ètica i Investigació Clínica de la Unió Catalana d’Hospitals* in accordance with the Code of Ethics of the World Medical Association (Declaration of Helsinki). Written informed consent was obtained from each participant prior to study enrollment.

### Experimental Set-Up

**FIGURE 1:**
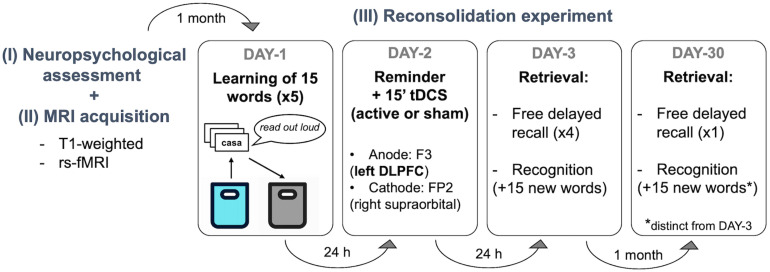
Experimental design. Participants visited our center 6 times in 2 months. The first visit was a (I) basic neuropsychological assessment followed by an (II) MRI acquisition session underwent during the same day or week. (III) The experimental protocol started one month later. There were 3 sessions separated by 24 h (DAY-1, DAY-2, DAY-3) and a follow-up assessment 1 month later (DAY-30). Verbal episodic memory learning was performed on DAY-1, stimulation was applied during 15 min after a contextual reminder on DAY-2, and delayed recall and recognition were measured 1 day after the stimulation session (DAY-3) and at 1-month follow-up (DAY-30). No information was given regarding the two retrieval sessions (i.e., DAY-3 and DAY-30). rs-fMRI, resting state functional resonance imaging; tDCS, transcranial direct current stimulations; DLPFC, left dorsolateral prefrontal cortex).

The present work was a randomized, double-blind, sham-controlled study. Participants and the study team members were not aware of the tDCS condition applied at any point of the experiment. Subjects were randomized into active or sham tDCS by using a simple randomization procedure with MATLAB (version R2019a, The MathWorks, Inc., Natick, MA, United States). The study protocol was accurately set based on previous publications (Manenti et al., 2017, 2018; Sandrini et al., 2019). The reconsolidation experiment *per se* began 1 month after the neuropsychological assessment (Supplementary Material Section 1.1) to avoid possible interferences with the verbal episodic memory test performed on the first visit (see Figure 1).

• DAY 1: learning session.

The episodic memory task was based on the Spanish-validated version of the Rey Auditory Verbal Learning Test (RAVLT; Schmidt, 1996). Subjects were instructed to try to remember as many words as possible from the cards that they had to transfer from one bag to another. Specifically, participants took each card (containing one written word) from a blue cloth bag, read them aloud, and put them in a black cloth bag. Following this, they were asked to recall as many words as possible. For the next learning trial, the 15 cards were placed in the blue bag again. This procedure was repeated five times as performed elsewhere (Manenti et al., 2017, 2018). At the end of this session, participants were asked to complete a memory strategies questionnaire (based on Manenti et al., 2010), which comprised 10 possible tactics commonly used to enhance the learning process.

• DAY-2: remainder + tDCS session

Twenty-four hours later, the same experimenter involved in DAY-1, within the same experimental room, encouraged participants to describe the procedure followed the day before. This was done by showing the blue and black cloth bags. Participants were interrupted if they started recalling any specific word. Between 5 and 10 min after this contextual reminder (Monfils et al., 2009), tDCS (active or sham) was administered (Sandrini et al., 2014; Manenti et al., 2017). Subjects completed a Visual Analog Scale (VAS; Crichton, 2001) mood questionnaire immediately before and after tDCS induction. Also, at the end of the stimulation session, participants were asked to complete a NIBS-related adverse events questionnaire (adapted from Brunoni et al., 2011) to measure the perceived discomfort induced by tDCS.

• DAY-3 and DAY-30: retrieval sessions

On DAY-3, the delayed recall was measured in 4 recorded and timed attempts (i.e., free delayed recall 1). Each attempt was alternated with a copy of 3 simple sets of figures to avoid verbal interference and subvocal repetition of words: (i) circle + overlapping rectangles and (ii) diamond + cube extracted from the Consortium to Establish a Registry for Alzheimer’s Disease (CERAD) neuropsychological battery, and (iii) the interlocking pentagons from MMSE. Following this, a timed recognition-task was conducted, where participants had to identify the 15 words from DAY 1, out of a total of 30 cards (i.e., recognition 1). At one-month follow-up (DAY-30) subjects returned and delayed recall was again measured with only a timed and recorded attempt (i.e., free delayed recall 2). Then, a second timed recognition-task was performed (i.e., recognition 2), with 30 cards of mixed words from DAY-1 and 15 new words (different set of cards from DAY-3). Of note, participants were never informed that words would be asked again on DAY-3 and DAY-30. In addition, at both retrieval sessions, the experimenter requested for any review of the words at home or any cognitive tasks (such as crosswords in-between experimental days) that might have caused either facilitation or interference. Furthermore, at DAY-30, participants underwent additional questionnaires to assess factors such as memory complaints and functional status: cognitive reserve (CR), memory complaints (MiCog), Cognitive Difficulty Scale (CDS), and Functional Activity Questionnaire Pfeffer (FAQ). Additionally, as sleep quality is linked to learning and memory processes (Diekelmann and Born, 2010), the Pittsburgh Sleep Quality Index (PSQI) was administered at DAY-3 and DAY-30.

### tDCS Parameters

Following the same procedure described in previous studies (Manenti et al., 2017, 2018; Sandrini et al., 2019), tDCS was applied at 1.5 mA through two saline-soaked sponge electrodes of 7 × 5 cm (current density: 0.043 mA/cm^2^; Antal et al., 2017). Stimulation was delivered using a DC-Stimulator Plus (neuroConn GmbH, Ilmenau, Germany) and the same montage was used in both experimental groups (active and sham). According to the international 10–10 system of measurement, the anodal electrode was positioned over the F3 (l-DLPFC) and the cathodal electrode was placed over the FP2 (right supraorbital area). In all groups, the current was initially increased and finally decreased in a ramp-like fashion of 10 s. In the sham condition, the current delivery was terminated after 15 s of stimulation with no further blinding processes. In the active group, the current was supplied for 15 min. The stimulation was applied with a double-blind fashion and all the parameters adhered to safety criteria guidelines (Rossi et al., 2009; Antal et al., 2017).

### MRI Acquisition Parameters

MRI data were acquired in a 3T Siemens scanner (MAGNETOM Prisma) with 32-channel head coil at the *Unitat d’Imatge per Ressonància Magnètica IDIBAPS (Institut d’Investigacions Biomèdiques August Pi i Sunyer)* at *Hospital Clínic de Barcelona*, Barcelona.

For all participants, a high-resolution T1-weighted structural image was obtained with a magnetization prepared rapid acquisition gradient-echo (MPRAGE) three-dimensional protocol [repetition time (TR) = 2,400 ms, echo time (TE) = 2.22 ms, inversion time = 1,000 ms, field of view (FOV) = 256 mm, 0.8-mm isotropic voxel]. They also underwent resting-state fMRI (rs-fMRI) multiband (anterior-posterior phase-encoding; acceleration factor = 8) interleaved acquisitions (T2^∗^weighted EPI scans, TR = 800 ms, TE = 37 ms, 595 volumes, 72 slices, slice thickness = 2 mm, FOV = 208 mm). All the MRI images were examined by a senior neuroradiologist [N.B.] for any clinically significant pathology (none found) and all study participants had Fazekas Scale scores lower than 1. Then, all acquisitions were visually inspected before analysis by one of the first co-authors [L.M.-P.] to ensure that they did not contain MRI artifacts or excessive motion.

### MRI Analyses

#### Cortical Thickness (CTh) Measures

Structural T1-weighted images were automatically processed with FreeSurfer (version 6.0)^1^ in order to obtain maps of CTh, calculated as the distance between the white and gray matter surfaces at each vertex of the reconstructed cortical mantle (Fischl and Dale, 2000). First, the images were processed individually, and the results were inspected visually to ensure the accuracy of registration, skull stripping, segmentation, and cortical surface reconstruction. Previous to statistical analysis, CTh maps were smoothed using a 2D Gaussian kernel of 15 mm full-width at half maximum (FWHM). We then carried out vertex-wise General Lineal Models (GLM) as implemented in FreeSurfer in order to study: (i) group differences in CTh between sham and active-tDCS subjects; (ii) group (active vs. sham) interaction in the correlation between CTh and episodic memory scores and; (iii) group differences among the active-tDCS group in order to compare responders and non-responders subgroups (see section “tDCS Effects in Episodic Memory Performance” for the sample stratification details). The resulting vertex-wise statistical maps were considered significant at *p* < 0.05 level. Maps were further corrected for family-wise error (FWE) using a Monte Carlo Null-Z simulation, with 10,000 repetitions and a cluster *p* < 0.05.

#### Functional Connectivity (FC) Measures

The FMRIB Software Library (FSL, version 5.0.11)^2^ and the Analysis of Functional NeuroImages (AFNI) were used for preprocessing and analyzing rs-fMRI data. Preprocessing pipeline and head movement considerations are described in Supplementary Material Section 1.2. The rs-fMRI analysis to obtain a rs-FC average strength measure of the focused resting-state network (RSN), namely the DMN [both its dorsal (dDMN) and its ventral (vDMN) components], and of two control systems, namely the sensorimotor (SMN) and the primary visual (PVN) networks, was performed using a dual-regression approach implemented in FSL (Stagg et al., 2014; Bächinger et al., 2017), and considering previously published networks (Shirer et al., 2012). Briefly, in the dual-regression approach, a spatial regression of the preprocessed data using the available RSN maps was computed to identify the time-course of the RSN for each subject. Second, a temporal regression with those time-courses was determined to get the subject-specific map of each RSN. The resulting subject-specific component maps were masked by the corresponding RSN. The mean value of the parameter estimates within each RSN mask was extracted for each individual subject. The average of this parameter can be considered as a metric of the average strength of rs-FC within each RSN. This analysis was performed for each RSN separately, and the networks of interest were considered in the subsequent statistical analyses (Bächinger et al., 2017). Furthermore, these rs-FC average strength measures were obtained for the sub-networks of each RSN for further exploratory analyses (see section “Statistical Analyses”).

### Statistical Analyses

Statistical analyses were performed using IBM SPSS Statistics (Statistical Package for Social Sciences, version 24.0. Armonk, NY: IBM Corporation). Demographic, and neuropsychological variables, functional assessment (i.e., CDS, MiCog, Pfeffer FAQ), depression measures (i.e., HDRS), CR and quality of sleep (i.e., PSQI) questionnaires, VAS scores, tDCS aftereffects, quality of sham, strategies used during the verbal episodic memory task, review the words learned at home, and possible interfering cognitive tasks, were compared between groups using independent-sample *t*-test. For categorical data, differences between groups were evaluated using the chi-squared (χ^2^)-test.

As regards the verbal episodic memory task, performance was measured as accuracy scores (hits minus intrusions) in DAY-1 (5 trials mean), DAY-3 (4 trials mean) and DAY-30 (1 trial), and, recognition scores (total hits minus intrusions) at DAY-3 and DAY-30. Repeated measures ANOVA was used to investigate differences between group trajectories (DAY-1 vs. DAY-3; DAY-3 vs. DAY-30; DAY-1 vs. DAY-30) in retrieval accuracy, recognition, time of retrieval and time of recognition. As *post-hoc* pairwise analyses, differences between tDCS conditions (active vs. sham) were performed with independent-sample *t*-test at each time-point (DAY-1; DAY-3; DAY-30). Likewise, differences between time-points (DAY-1 vs. DAY-3; DAY-3 vs. DAY-30; DAY-1 vs. DAY-30) for each group (active and sham independently) were measured using paired-samples *t*-tests.

Similar to the neuroimaging approach, we tested differences between responder and non-responder subgroups among the active-tDCS group (see section “tDCS Effects in Episodic Memory Performance” for the sample stratification details), in order to identify baseline cognitive and/or demographic characteristics associated with a greater tDCS-related modulation. Further, the rs-FC average strength measures obtained [see section “Functional Connectivity (FC) Measures”] were compared between subgroups (responder vs. non-responder). In addition, these rs-FC values were used to test correlations with the cognitive change.

Data distribution was tested for normality with Shapiro-Wilk test (*p* > 0.05). For those variables not normally distributed, non-parametric tests were used: Mann-Whitney U was carried out for group comparisons and Spearman correlations were performed to test correlations. Differences were considered significant at *p* < 0.05. Graphical representations were performed using IBM SPSS Statistics and GraphPad Prism (version 6.00, GraphPad Software, La Jolla, CA, United States).

## Results

### Sample Characterization

No baseline differences were found between sham and active tDCS groups for demographic variables, neuropsychological and functional assessment, depression, CR and sleep quality. It should be noted that the number of on-treatment controlled hypertension, diabetes, and hyperlipidemia in both groups were equivalent (for further details see Supplementary Table 1). There were no group differences regarding the use of strategies during the verbal episodic memory task (χ^2^ = 1.027, *p* = 0.311), reviewing the learned words at home (DAY-3: χ^2^ = 0.792, *p* = 0.374; DAY-30: χ^2^ = 0.175, *p* = 0.676) or performing possible interfering cognitive tasks (χ^2^ = 0.230, *p* = 0.631). Furthermore, VAS pre-stimulation scores (all *p* > 0.05, see Supplementary Table 2) and quality of sham questionnaire results (χ^2^ = 1.250, *p* = 0.535) did not differ between groups. All participants tolerated the stimulation well, and only heat and pinching (categorized as mild or moderate) were reported as tDCS-related adverse events in a similar proportion for both groups (heat: χ^2^ = 1.254, *p* = 0.534; pinching: χ^2^ = 1.067, *p* = 0.587).

### tDCS Effects in Episodic Memory Performance

**FIGURE 2:**
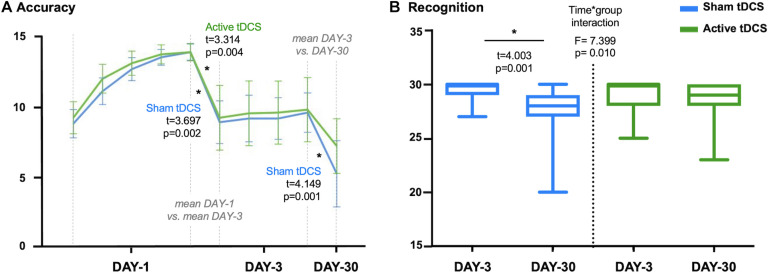
**(A)** Accuracy scores for sham-tDCS and active-tDCS groups at each experimental session: five attempts on first session (DAY-1), four attempts on third session (DAY-3) and one attempt on follow-up session (DAY-30). Note that differences between measures are calculated considering the mean accuracy at DAY-1 and DAY-3. **(B)** Plot showing the time*group interaction in recognition. The sham-tDCS group showed a significant decline after 1-month follow-up, while there was not a significant difference between DAY-3 and DAY-30 for the active-tDCS group. tDCS, transcranial direct current stimulations. *p < 0.05.

Episodic memory performance decreased with time for the whole sample (DAY-1 vs. DAY-3: *t* = 4.934, *p* < 0.001; DAY-3 vs. DAY-30: *t* = 4.164, *p* < 0.001). Both active and sham groups significantly reduced accuracy performance on DAY-3 as compared with DAY-1 (active: *t* = 3.314, *p* = 0.004; sham: *t* = 3.697, *p* = 0.002). Critically, the active group maintained episodic memory accuracy in the follow-up session (DAY-30) compared to DAY-3 (*t* = 1.929, *p* = 0.070), while the sham group significantly decreased the number of words retrieved at DAY-30 (*t* = 4.149, *p* = 0.001; see Figure 2A). However, no significant group differences were detected at any experimental session as regards retrieval accuracy (DAY-1: *t* = 1.094, *p* = 0.281; DAY-3: *t* = 0.153, *p* = 0.879; DAY-30: *t* = 1.352, *p* = 0.185).

Focusing on recognition scores, we identified a time^∗^group interaction (*F* = 7.399, *p* = 0.010). Specifically, performance in recognition significantly declined between the third session (DAY-3) and the 1-month follow-up measure (DAY-30) for the sham group (*t* = 4.003, *p* = 0.001). On the other hand, the active group maintained a stable recognition performance across measures (*t* = 0.900, *p* = 0.380; see Figure 2B). However, there were no significant differences between groups in recognition at DAY-3 (*t* = 1.168, *p* = 0.250) or DAY-30 (*t* = −1.686, *p* = 0.100).

There were no group differences regarding the time spent for completing the learning session (DAY-1: *t* = 0.992, *p* = 0.328), the retrieval (DAY-3: *t* = 0.848, *p* = 0.402; DAY-30: *t* = 0.058, *p* = 0.954), or recognition (DAY-3: *t* = 0.634, *p* = 0.530; DAY-30: *t* = −0.416, *p* = 0.680). There were no significant time^∗^group interactions considering DAY-3 and DAY-30 time-related scores (retrieval: *F* = 0.429, *p* = 0.517; recognition: *F* = 0.684, *p* = 0.414). However, the active-tDCS group significantly increased the time employed for the recognition task at DAY-30 compared with DAY-3 (*t* = −2.585, *p* = 0.019). It should be noted that the time^∗^group interaction identified for the recognition performance between DAY-3 and DAY-30 (Figure 2B) remained significant after adjusting by time of recognition (data not shown). For further details regarding episodic memory performance see Supplementary Table 3.

Finally, due to the tDCS effect in recognition (Figure 2B), we split the active group into responders and non-responders according to the difference between DAY-3 and DAY-30 in recognition scores. As a result of this, 13 out of 19 subjects were classified as responders (change equal or above 0; i.e., stable progression) and 6 out of 19 as non-responders (change below 0; i.e., longitudinal decline). We did not identify differences between these subgroups regarding demographic variables, clinical data (i.e., hypertension, diabetes, hyperlipidemia), other questionnaires (i.e., CDS, MiCog, Pfeffer FAQ, CR, PSQI), or cognitive performance (considering the neuropsychological evaluation and memory scores at DAY-1).

### MRI-Based Measures Related to tDCS Responsiveness

#### Cortical Thickness

**FIGURE 3:**
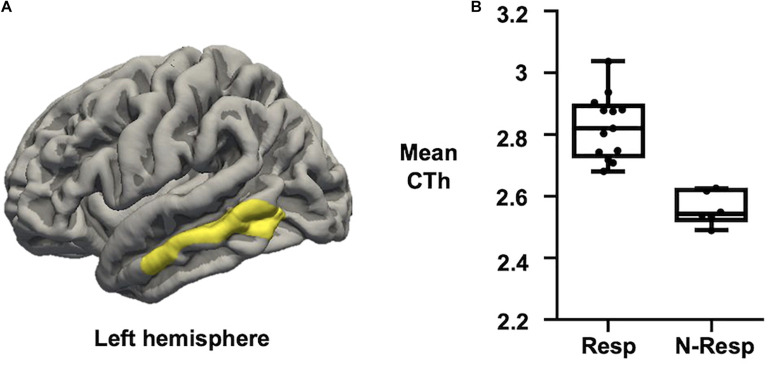
**(A)** Map of CTh showing that active-tDCS responders exhibited thicker left middle temporal lobe than active-tDCS non-responders. Only clusters surviving FWE multiple comparison correction with a final cluster-wise *p* < 0.05 are considered. **(B)** Plot showing that Resp exhibited higher CTh than N-Resp (mean CTh values extracted from the significant cluster over the left middle temporal lobe). CTh, cortical thickness; FWE, family-wise error; Resp, responders; N-Resp, non-responders.

Regarding CTh measures, we first confirmed no significant differences between sham and active groups. Afterward, when only considering those subjects who received active-tDCS, significant differences were detected comparing responders vs. non-responders. More specifically, those participants classified as responders exhibited greater CTh than non-responders in a cluster placed over the left middle temporal lobe (see Figure 3). No significant associations were observed between recognition change scores and CTh measures.

#### Resting-State Functional MRI

**FIGURE 4:**
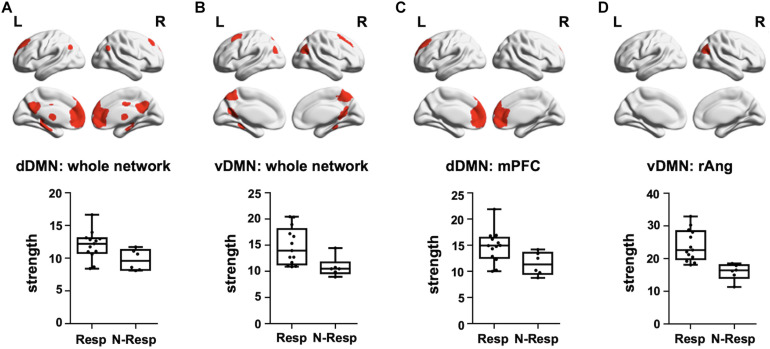
Top—Brain masks covering the **(A)** dDMN, **(B)** vDMN, **(C)** medPref ROI within the dDMN, and **(D)** rAng ROI within the vDMN, are shown in red on a standard template. Bottom—Graphical representation of strength values for each group (Resp and N-Resp) within the DMN-related areas of interest. dDMN, dorsal default mode network; vDMN, ventral default mode network; medPref, medial prefrontal cortex; rAng, right angular gyrus; Resp, Responders; Non-resp, Non-responders; L, left; R, right.

We first evidenced that the strength of the dDMN and the vDMN, as well as of the control networks (i.e., SMN and PVN), did not differ between sham and active groups (all *p* > 0.05). Subsequently, analyses were focused on the active-tDCS group and significant differences between responders and non-responders were detected on the two networks of interest (*p* < 0.05), but not in the control networks (*p* > 0.05). More precisely, responders showed greater rs-fMRI strength both in the dDMN (*t* = 2.264, *p* = 0.037) and the vDMN (*t* = 3.190, *p* = 0.006). As an exploratory analysis, we also investigated which specific sub-networks within the selected networks drove these differences. Thus, among the dDMN sub-networks, the rs-FC within the medial prefrontal (medPref) and the midcingulate cortices (midCC) were distinct between subgroups (medPref: *t* = 2.318, *p* = 0.033; midCC: *t* = 2.508, *p* = 0.023) in the same direction. Furthermore, the right angular gyrus (rAng) FC within the vDMN emerged also to be statistically different between subgroups in the abovementioned direction (*t* = 4.799; *p* < 0.001; see Figure 4). We conducted additional correlational analyses to corroborate with continuous data these results. In this vein, we assessed if the observed networks and sub-networks were also directly related to the recognition change scores in the active-tDCS group. Indeed, we observed a positive correlation between the difference in memory recognition on DAY-30 and DAY-3 and the rs-FC strength within the whole dDMN (ρ = 0.507, *p* = 0.027) and vDMN (ρ = 0.512, *p* = 0.025). Regarding the sub-networks, within the dDMN, we also observed significant associations with the medPref (ρ = 0.510, *p* = 0.026), but not with the midCC (*p* > 0.05). Within the vDMN, we also detected a significant correlation with the rAng region (ρ = 0.507, *p* = 0.027). We corroborated that none of those correlations were present in the sham group (all *p* > 0.05), being thus specific for the active-tDCS group.

## Discussion

The present study is, to the best of our knowledge, the first independent replication of a protocol aimed to improve memory recognition through the use of tDCS with a contextual reminder in SCD participants. Moreover, this study sheds light on the neural signatures underlying this memory modulation process, contributing to the understanding of brain structural and functional correlates subtending cognitive responsiveness to a specific tDCS procedure aimed to enhance episodic memory.

### Effects of tDCS on Episodic Memory Reconsolidation Strengthening

Regarding the behavioral effects, this investigation showed that anodal tDCS applied over the l-DLPFC is capable of strengthening existing episodic memories by reducing recognition performance loss up to 1 month, relative to sham stimulation, in individuals with SCD. Further, consistent with the previously achieved work (Manenti et al., 2017), we also identified clear tDCS-related cognitive effects on recognition, but not on free recall. This is in line with previous evidence showing that the familiarity component of recognition is relatively preserved in the aging process, whereas recollection does show age-related loss (Danckert and Craik, 2013). Moreover, no group differences were detected on the learning curve and immediate free recall (DAY-3), which is consistent with the notion that certain tDCS effects might take place after rather than during stimulation (Flöel et al., 2012; Abellaneda-Pérez et al., 2020). Anatomically, the current work and previous reports in the field (reviewed in Sandrini et al., 2020) are in accordance with the claim that l-DLPFC might have a causal role in the strengthening of existing episodic memories in a reconsolidation framework. Beyond the importance of this replication in terms of beneficial effects of tDCS on memory function, the present findings support the view that the memory reconsolidation process is susceptible to be strengthened by applying tDCS over the l-DLPFC after a contextual remainder (Sandrini et al., 2014, 2019; Manenti et al., 2017).

### Structural and Functional MRI Characteristics Related to tDCS Effects

Concerning the neural basis of the tDCS-induced cognitive effects, our findings suggested that responsiveness to brain stimulation in this population could be partially explained by differences in structural and functional brain characteristics, as previous research suggested (Kim et al., 2014; Abellaneda-Pérez et al., 2018). At a structural level, CTh differences detected between cognitive responders and non-responders among the active group over the left middle temporal cortex are in line with the hypothesis that individual variability in behavioral outcomes of tDCS might be partly explained by individual anatomical differences (Kim et al., 2014). In particular, our results suggest that greater structural integrity in key memory-related areas (i.e., middle temporal lobe) predicted better cognitive gains induced by tDCS. Further, we assessed functional connectivity to evaluate if individual cognitive responsiveness to the tDCS-reconsolidation approach was predicted by rs-fMRI measures. We found that DMN intrinsic connectivity was associated with the magnitude of individual tDCS-induced memory enhancement in recognition. These results support that an adequate memory functioning, and particularly, a NIBS-induced enhancement in this cognitive domain, also depends on the integrity of brain functional systems associated with preserved cognitive functioning in aging (Abellaneda-Pérez et al., 2018; Antonenko et al., 2019). Therefore, the integrity of the DMN, which is crucial in cognitive aging and in SCD (Wang et al., 2013), is also critical to determine the individual capability to respond to stimulation protocols aimed to modulate cognition (Vidal-Piñeiro et al., 2015; Abellaneda-Pérez et al., 2018; Antonenko et al., 2019). Overall, these MRI-derived results highlight that a detailed subject-specific brain characterization might be used in forthcoming trials to select those individuals that may benefit the most from NIBS-based interventions. Finally, it is relevant to consider that the responder and non-responder groups classified following our tDCS-reconsolidation protocol, which entailed structural and functional neuroimaging differences, did not differ in baseline cognition, demographic variables, or clinical data. Thus, the stated imaging group differences are not likely explained by potential dissimilarities regarding vascular risk factors, which might have implied potential cortical excitability differences in response to NIBS, as previously reported (reviewed in Cantone et al., 2020).

### Limitations

Despite the relatively small number of participants could represent a limitation, the sample was larger than previous similar studies (Sandrini et al., 2014, 2019; Manenti et al., 2017, 2018). However, future research applying multiple sessions of tDCS after the contextual reminder should be conducted to define the potentially long-lasting effects of this non-invasive intervention. The present neuroimaging findings suggesting that greater structural and functional brain preservation may represent a key factor in the selection of those individuals with greater chances of interventional success might be efficiently used to boost the global outcomes of cognitive-NIBS therapeutical approaches in individuals with self-perceived cognitive decline. Notwithstanding, it should be noted that these brain-behavioral associations are only linked to the specific protocol used, and thus, our data may also suggest that distinct cognitive settings or different stimulation doses might be more appropriate. In the same line, further studies are needed to determine optimal stimulation designs and to shed light on the specific neurobiological mechanisms underlying the cognitive effects induced by this intervention before conducting clinical trials applying this memory reconsolidation protocol with tDCS.

## Conclusion

In conclusion, this study is a research group-independent replication of Manenti et al. (2017) with a larger sample indicating that anodal tDCS over the l-DLPFC during reconsolidation can improve memory amongst participants with SCD up to 1 month. Thus, this cognitive-NIBS approach entails clear potential to feasibly reduce memory loss in subjects at risk for AD. This investigation represents a major milestone in the development of effective cognitive interventions in populations both healthy and referring memory deficits. In addition, our findings highlight that specific brain characteristics are valuable to understand and predict individual stimulation effects. Within those neural features, structural preservation of temporal lobe regions and functional integrity of the default-mode system appears to be critical to determine the cognitive responsiveness to electrical stimulation in subjects with SCD.

## Data Availability Statement

The raw data supporting the conclusions of this article will be made available by the authors, without undue reservation.

## Ethics Statement

The studies involving human participants were reviewed and approved by the University of Barcelona’s Bioethics Commission (CBUB). The patients/participants provided their written informed consent to participate in this study.

## Author Contributions

LV-A, KA-P, CS-P, and DM contributed to the conception and design of the study. LM-P, GC, and JS organized the database. LV-A, LM-P, KA-P, and MC-T analyzed the data. LV-A and LM-P wrote the first draft of the manuscript. KA-P, MC-T, and DM wrote sections of the manuscript. All authors contributed to manuscript revision, read, and approved the submitted version.

## Conflict of Interest

AP-L was listed as an inventor on several issued and pending patents on the real-time integration of non-invasive brain stimulation with electroencephalography and magnetic resonance imaging. He was co-founder of Linus Health and TI Solutions AG; serves on the scientific advisory boards for Starlab Neuroscience, Magstim Inc., Nexstim, and MedRhythms, and is an Associate Editor for Annals of Neurology. AP-L serves on the scientific advisory boards for Starlab Neuroscience, Neuroelectrics, Axilum Robotics, Constant Therapy, NovaVision, Cognito, Magstim, Nexstim, and Neosync, and is listed as an inventor on several issued and pending patents on the real-time integration of transcranial magnetic stimulation with electroencephalography and magnetic resonance imaging. The remaining authors declare that the research was conducted in the absence of any commercial or financial relationships that could be construed as a potential conflict of interest.

## Publisher’s Note

All claims expressed in this article are solely those of the authors and do not necessarily represent those of their affiliated organizations, or those of the publisher, the editors and the reviewers. Any product that may be evaluated in this article, or claim that may be made by its manufacturer, is not guaranteed or endorsed by the publisher.

## Footnotes

1 https://surfer.nmr.mgh.harvard.edu

2 https://fsl.fmrib.ox.ac.uk/fsl/fslwiki/
