# Supplementary material for: tDCS-Induced Memory Reconsolidation Effects and Its Associations With Structural and Functional MRI Substrates in Subjective Cognitive Decline
Source: Front Aging Neurosci. 2021 Jul 26;13:695232. doi: 10.3389/fnagi.2021.695232 (PMC8350070; doi:10.3389/fnagi.2021.695232)
Supplement: Supplementary file 1 [file Table_1.pdf]

Supplementary Material for:

tDCS-induced memory reconsolidation effects and its associations with structural and functional MRI substrates in subjective cognitive decline.

Lídia Vaqué-Alcázar[1,2†] (PhD), Lídia Mulet-Pons[1,2†] (MSc), Kilian Abellaneda-Pérez[1,2] (PhD), Cristina Solé-Padullés[1] (PhD), María Cabello-Toscano[1,2,3] (MSc), Dídac Macià[4] (PhD), Roser Sala-Llonch[2,4,5] (PhD), Nuria Bargalló[2,6] (MD, PhD), Javier Solana[3] (PhD), Gabriele Cattaneo[2,3] (PhD), José M. Tormos[3] (MD, PhD), Alvaro Pascual-Leone[3,7] (MD, PhD), David Bartrés-Faz[1,2,3] (PhD).

[1] Department of Medicine, Faculty of Medicine and Health Sciences, Institute of Neurosciences, University of Barcelona, 08036, Barcelona, Spain.

[2] Institut de Recerca Biomèdica August Pi i Sunyer (IDIBAPS), 08036, Barcelona, Spain.

[3] Guttmann Institute, Badalona, Barcelona, Spain

[4] Department of Biomedicine, Faculty of Medicine and Health Sciences, Institute of Neurosciences, University of Barcelona, 08036, Barcelona, Spain.

[5] Consorcio Centro de Investigación Biomédica en Red (CIBER) de Bioingeniería, Biomateriales y Nanomedicina (CIBER-BBN), Barcelona, Spain

[6] Centre de Diagnòstic per la Imatge Clínic, Hospital Clínic de Barcelona, 08036, Barcelona, Spain.

[7] Hinda and Arthur Marcus Institute for Aging Research and Deanna and Sidney Wolk Center for Memory Health, Hebrew SeniorLife, Harvard Medical School, Boston, MA, United States of America.

[†]These authors have contributed equally to this work and share first authorship

Correspondence

Lídia Vaqué-Alcázar

Tel: +34 934039295

E-mail: lidiavaque@ub.edu

David Bartrés-Faz

Tel: +34 934039295

E-mail: dbartres@ub.edu

Department of Medicine, Faculty of Medicine and Health Sciences, University of Barcelona.

Casanova, 143, 08036, Barcelona, Spain.

1. Supplementary Methods

1.1. Neuropsychological assessment

On the first visit to our center (one month before the experimental protocol) each subject underwent a battery of neuropsychological tests assessing the main cognitive domains. Previously, the Mini-Mental State Examination was administered for screening purposes (MMSE; Lobo et al., 1980). Neuropsychological tests included: Free and Cued Selective Reminding test, Rey-Osterrieth Complex Figure, Digit Span forward and backward, Trial Making Test A and B, Visual Object and Space Perception, Symbol Digit Modalities Test, Phonetic and semantic fluency tests and Boston Naming Test. Normalized scalar scores NEURONORMA Project for Spanish population were used for all tests included in the assessment (Peña-Casanova et al., 2009). The Vocabulary subtest from the WAIS-IV (Wechsler 2008) was also administered. Depressive symptoms were assessed with the Hamilton Depression Rating Scale (HDRS; Lobo et al., 2002).

1.2. fMRI analyses

1.2.1. Preprocessing

The functional magnetic resonance (fMRI) preprocessing pipeline made use of functions from FMRIB Software Library (FSL; version 5.0.11; https://fsl.fmrib.ox.ac.uk/fsl/fslwiki/), FreeSurfer (version 6.0; https://surfer.nmr.mgh.harvard.edu) and Statistical Parametric Mapping (SPM12; https://www.fil.ion.ucl.ac.uk/spm/). To start with, the first 10 scans were removed to ensure magnetization equilibrium. After that, all images were field inhomogeneity corrected (*FSL topup tool*), realigned to a reference image (*FSL MCFLIRT*) and standardized into native T1-weighed space (*SPM Coregister*). Signals from white matter (WM) and cerebrospinal fluid (CSF) were extracted and considered as nuisance regressors.

Tissue segmentation, for the obtention of these CSF and WM masks, was part of the FreeSurfer automatic processing of T1-weighted images, already detailed in the main manuscript (MATERIALS AND METHODS section). Other regressors were those correspondent to motion and to a drift of low frequency oscillations. Motion regressors were 12 and were estimated during the realignment; six were those of rotation and translation and the other six were their first derivative. The drift was estimated by a discrete cosine transform (DCT) as a low-pass frequency filter (<0.01). All these regressors were regressed out using the *fsl_regfilt* tool implemented in FSL. Finally, normalization (*SPM Normalize*) of all fMRI images to Montreal Neuroscience Institute (MNI152) standard space was performed to ensure among-subjects comparability.

1.2.2. Head motion correction

As head movement may affect rs-fMRI results (Power et al., 2012, 2015; Van Dijk et al. 2012), in-scanner head motion was considered. In this study, the frame-wise displacement (FWD) mean was calculated for every subject. FWD was computed as in Power et al. (2012). In our sample, no significant differences as regards this measure were found between the two experimental groups (i.e., active- vs sham-tDCS) neither between responders vs. non-responders within the active-tDCS group ($p > 0.05$).

2. Demographic variables, memory task performance and questionnaires scores

	All sample (N=38)	Active tDCS (N=19)	Sham tDCS (N=19)	*p-value*
**Demographics**				
Gender (male/female)	19/19	8/11	11/8	0.330 [a]
Age	62.29 (1.56)	62.58 (1.43)	62 (1.67)	0.210 [b]
Years of education	12.37 (2.24)	12.31 (2.36)	12.42 (2.17)	0.988 [b]
CR questionnaire	16.05 (3.50)	16.68 (3.28)	15.42 (3.67)	0.272 [c]
**Screening**				
MMSE	28. 95 (1.21)	28.74 (1.24)	29.16 (1.17)	0.142 [b]
HDRS	2.94 (2.63)	3.26 (2.86)	2.63 (2.40)	0.625 [b]
**Functional Assessment**				
CDS	40.18 (16.27)	42.58 (18.44)	37.79 (13.86)	0.693 [c]
MiCog	9.84 (3.57)	10.68 (3.13)	9 (3.86)	0.148 [c]
Pfeffer FAQ	0.34 (0.58)	0.42 (0.69)	0.26 (0.45)	0.593 [b]
**Global cognition**				
Vocabulary (WAIS-IV)	32.13 (7.43)	33.32 (7.45)	30.95 (7.42)	0.254 [b]
**Memory**				
FCSRT immediate-free	27.79 (6.47)	28.16 (6.10)	27.42 (6.96)	0.731 [c]
FCSRT immediate-facilitated	40.31 (6.19)	40.53 (5.58)	40.10 (6.89)	0.977 [b]
FCSRT delayed-free	11.92 (2.17)	12.26 (2.51)	11.58 (1.77)	0.339 [c]
FCSRT delayed-facilitated	15.34 (0.70)	15.47 (0.61)	15.21 (0.79)	0.298 [b]
RCF immediate recall	20.60 (5.84)	20.60 (5.97)	20.60 (5.87)	1.000 [c]
RCF delayed recall	18.64 (8.23)	16.74 (9.02)	20.55 (7.08)	0.148 [b]
**Attention**				
Digit span forward	9 (2.08)	9.21 (2.37)	8.79 (1.78)	0.282 [b]
TMT-A	33.13 (9.04)	31.47 (8.55)	34.79 (9.44)	0.254 [b]
**Praxis**				
RCF copy	34.54 (0.24)	34.13 (2.80)	34.95 (1.47)	0.649 [b]

**Visuospatial function**				
VOSP (numbers)	9.42 (0.69)	9.58 (0.50)	9.26 (0.80)	0.229 [b]
**Executive functions**				
Digit span backward	6.71 (1.77)	6.84 (2.00)	6.58 (1.54)	0.514 [b]
TMT-B	72.42 (20.60)	68.16 (21.60)	76.68 (19.16)	0.068 [b]
SDMT	46.73 (10.10)	46.53 (12.22)	46.93 (7.76)	0.726 [b]
**Language**				
Phonetic fluency (F-A-S)	39.55 (8.81)	40.37 (9.87)	38.73 (7.80)	0.650 [c]
Semantic fluency (animals)	20.97 (4.55)	21.31 (4.88)	20.63 (4.3)	0.660 [b]
BNT	55.38 (5.14)	54.33 (6.47)	56.42 (3.20)	0.201 [b]
**Sleep Quality**				
PQSI (DAY-3)	5.74 (3.85)	4.89 (3.75)	6.58 (3.86)	0.181 [c]
PQSI (DAY-30)	4.79 (3.02)	4.26 (3.30)	5.31 (2.67)	0.290 [b]
**Clinical Data**				
Hypertension (Yes/No)	7/31	3/16	4/15	0.693 [d]
Diabetes (Yes/No)	2/36	0/19	2/17	0.230 [d]
Hyperlipidemia (Yes/No)	4/34	1/18	3/16	0.640 [d]

Supplementary Table 1. Sample characteristics. Data are presented as mean (standard deviation) for the whole sample and taking into account each group (active and sham).
[a] *p*-values were obtained by χ^2 test (categorical data).
[b] *p*-values were obtained by Wilcoxon Mann Whitney u-test (non-parametric data).
[c] *p*-values were obtained by two sample t-test (parametric data).
[d] *p*-values were obtained by Fisher's exact test.
Abbreviations: tDCS, transcranial direct current stimulation; CR, Cognitive Reserve; MMSE, Mini-Mental State Examination; HDRS, Hamilton Depression Rating Scale; CDS, Cognitive Difficulty Scale; Pfeffer FAQ, Pfeffer Functional Activities Questionnaire; FCSRT, Free and Cued Selective Reminding test; RCF, Rey Complex Figure; TMT, Trial Making Test; VOSP, Visual Object and Space Perception; SDMT, Symbol Digit Modalities Test; BNT, Boston Naming Test; PSQI, Pittsburgh Sleep Quality Index.

	**All sample (N=38)**	**Active tDCS (N=19)**	**Sham tDCS (N=19)**	***p*-value**
**Strategies use**				
DAY-1	37/38	19/19	18/19	0.311 [a]
**Review words**				
DAY-3	6/38	4/19	2/19	0.374 [a]
DAY-30	7/38	3/19	4/19	0.676 [a]
**Interfering task**				
DAY-3	5/38	3/19	2/19	0.631 [a]
**VAS pre tDCS**				
Contentment	6.66 (1.83)	6.92 (1.87)	6.40 (1.80)	0.220 [b]
Sadness	1.12 (1.23)	0.97 (0.72)	1.26 (1.60)	0.753 [b]
Hope	6.53 (1.90)	7.08 (1.84)	5.97 (1.83)	0.094 [b]
Annoyance	1.89 (2.70)	1.82 (2.46)	1.97 (2.99)	0.854 [b]
Nervousness	1.91 (2.13)	2.10 (2.53)	1.71 (1.70)	0.965 [b]
**VAS post tDCS**				
Contentment	6.54 (1.78)	6.87 (1.76)	6.21 (1.78)	0.260 [c]
Sadness	1.26 (1.55)	1.32 (1.58)	1.21 (1.55)	0.687 [b]
Hope	6.40 (1.96)	6.84 (1.92)	5.95 (1.94)	0.201 [b]
Annoyance	1.88 (2.40)	1.58 (2.00)	2.18 (2.76)	0.522 [b]
Nervousness	1.53 (1.72)	1.68 (1.92)	1.37 (1.54)	0.882 [b]
**Quality of sham**				
DAY-2	18/38	9/19	9/19	0.535 [a]
**Adverse effects tDCS**				
Heat	10/38	6/19	4/19	0.311 [a]
Pinching	18/38	9/19	9/19	0.819 [a]

Supplementary Table 2. Additional data of stimulation application and episodic memory task. Data are presented as mean (standard deviation) for the whole sample and taking into account each group (active and sham). There are not mood variations due to stimulation in none of the two groups, there are not significant differences between pre- and post-stimulation VAS scores in the all sample, active and sham group.

[a] *p*-values were obtained by χ^2 test (categorical data)

[b] *p*-values were obtained by Wilcoxon Mann Whitney u-test (non-parametric data)

[c] *p*-values were obtained by two sample t-test (parametric data)

Abbreviations: VAS, Visual Analogue Scale; tDCS, transcranial direct current stimulation.

	All sample (N=38)	Active tDCS (N=19)	Sham tDCS (N=19)	*p*-value
**Accuracy**				
DAY-1	11.74 (1.84)	12.06 (1.78)	11.41 (1.90)	0.281 [a]
DAY-3	8.97 (4.19)	9.08 (5.06)	8.87 (3.24)	0.879 [a]
DAY-30	6.20 (4.19)	7.18 (4.05)	5.21 (4.90)	0.185 [a]
**Recognition**				
DAY-3	29.13 (1.25)	28.90 (1.49)	29.37 (0.95)	0.377 [b]
DAY-30	28 (1.25)	28.58 (1.86)	27.42 (2.34)	0.040* [b]
**Time of encoding**				
DAY-1	75.65 (14.81)	73.26 (11.70)	78.03 (17.39)	0.329 [a]
**Time of retrieval**				
DAY-3	82.25 (34.02)	77.55 (29.37)	86.95 (38.33)	0.402 [a]
DAY-30	104.05 (38.47)	103.68 (42.72)	104.42 (34.89)	0.954 [a]
**Time of recognition**				
DAY-3	73.68 (36.02)	69.95 (38.30)	77.42 (34.20)	0.530 [a]
DAY-30	93.58 (37.02)	96.11 (37.73)	91.05 (37.16)	0.680 [a]

Supplementary Table 3. Verbal episodic memory task scores. Data are presented as mean (standard deviation) for the whole sample and taking into account each group (active and sham) at each time-point measure (DAY-1, DAY-3 and DAY-30). Note that units for time measures are seconds.

* $p < 0.05$.

[a] *p*-values were obtained by two sample t-test (parametric data)

[b] *p*-values were obtained by Wilcoxon Mann Whitney u-test (non-parametric data)

Abbreviations: tDCS, transcranial direct current stimulation.

REFERENCES

Lobo, A., Escobar, V., Ezquerra, J., and Seva Díaz, A. (1980). "El Mini-Examen Cognoscitivo" (Un test sencillo, práctico, para detectar alteraciones intelectuales en pacientes psiquiátricos) [The "Mini-Examen Cognoscitiuo": A simple and practical test to detect intellectual dysfunctions in psychiatric patients]. Revista de Psiquiatría y Psicología Médica 14(5),39–57.

Lobo, A., Chamorro, L., Luque, A., Dal-Ré, R., Badia, X., and Baró E. (2002). Validación de las versiones en español de la Montgomery-Asberg Depression Rating Scale y la Hamilton Anxiety Rating Scale para la evaluación de la depresión y de la ansiedad [Validation of the Spanish versions of the Montgomery-Asberg depression and Hamilton anxiety rating scales]. Med Clin. 118(13),493-499. doi:10.1016/s0025-7753(02)72429-9

Peña-Casanova, J., Blesa, R., Aguilar, M., Gramunt-Fombuena, N., Gómez-Ansón, B., Oliva, R., et al. (2009). Spanish Multicenter Normative Studies (NEURONORMA Project): methods and sample characteristics. Arch Clin Neuropsychol. 24(4),307-319. doi:10.1093/arclin/acp027

Power, J.D., Barnes, K.A., Snyder, A.Z., Schlaggar, B.L., and Petersen, S.E. (2012). Spurious but systematic correlations in functional connectivity MRI networks arise from subject motion. Neuroimage 59(3),2142-2154. doi:10.1016/j.neuroimage.2011.10.018

Power, J.D., Schlaggar, B.L., Petersen, S.E. (2015). Recent progress and outstanding issues in motion correction in resting state fMRI. Neuroimage 105,536-551. doi:10.1016/j.neuroimage.2014.10.044

Van Dijk, K.R., Sabuncu, M.R., Buckner, R.L. (2012). The influence of head motion on intrinsic functional connectivity MRI. Neuroimage 59(1),431-438. doi:10.1016/j.neuroimage.2011.07.044

Wechsler, D. (2008). Wechsler adult intelligence scale–Fourth Edition (WAIS–IV). San Antonio, TX: NCS Pearson 22(498),1.
